# Providing Verbal Assistance When Assessing Individuals Living with a Traumatic Brain Injury

**DOI:** 10.1177/00084174211034263

**Published:** 2021-08-31

**Authors:** Mireille Gagnon-Roy, Nathalie Bier, Stéphanie Boulé-Riley, Heidi Keurentjes, Priscilla Lam Wai Shun, Guylaine Le Dorze, Carolina Bottari

**Keywords:** Activities of daily living, Case studies, Clinical reasoning, Cognition disorders, Activités de la vie quotidienne, études de cas, raisonnement clinique, troubles de la cognition

## Abstract

**Background.** Occupational therapists play a major role in identifying the assistance needs of individuals living with a traumatic brain injury. However, to obtain an accurate assessment, verbal assistance should be provided only when necessary, according to the person's needs. **Purpose.** This study aimed to understand (1) how verbal assistance is provided during an evaluation of Instrumental Activities of Daily Living and (2) why it is provided in this manner. **Method.** Interviews were conducted with three expert occupational therapists using their own videotaped evaluation and a “think-aloud” method to explore their clinical reasoning when providing verbal assistance. Data were analyzed using thematic analysis. **Findings.** The process of providing verbal assistance was recognized as flexible and nonlinear, and influenced by various factors including the participants’ level of understanding of the client's abilities. **Implications.** This information will help therapists better understand how and when to provide verbal assistance when assessing their clients.

## Abrégé

**Description.** Les ergothérapeutes jouent un rôle majeur dans l'identification des besoins des personnes vivant avec un traumatisme crânio-cérébral en termes d'assistance. Cependant, pour obtenir une évaluation précise, l'assistance verbale ne doit être fournie que lorsque cela est nécessaire, en fonction des besoins de la personne. **But.** Cette étude visait à comprendre (1) comment l'assistance verbale est fournie lors d'une évaluation des activités instrumentales de la vie quotidienne et (2) pourquoi elle est fournie de cette manière. **Méthodologie.** Des entretiens ont été menés avec trois ergothérapeutes experts en utilisant leur propre évaluation filmée et une méthode de « réflexion à voix haute » pour explorer leur raisonnement clinique lorsqu'ils fournissent de l'assistance verbale. Les données ont été analysées à l'aide d'une analyse thématique. **Résultats.** Offrir de l'assistance verbale a été reconnu comme étant un processus flexible et non linéaire, et influencé par divers facteurs, notamment le niveau de compréhension des participants quant aux capacités du client. **Conséquences.** Ces informations aideront les thérapeutes à mieux comprendre comment et quand fournir de l'assistance verbale lors de l'évaluation de leurs clients.

## Introduction

Living with a traumatic brain injury (TBI) can lead to major challenges with thinking through all of the decisions involved in completing complex everyday activities such as preparing meals, shopping for groceries or managing one's finances (Bottari et al., 2011; Fortin et al., 2003; Godbout et al., 2005; Lefebvre et al., 2008). In rehabilitation, occupational therapists (OTs) play a major role in assessing how specific cognitive deficits (e.g., executive functioning, attention, and memory) affect participation in meaningful activities (INESSS-ONF, 2016, section H.1.1) and in determining which interventions to prioritize according to the observed cognitive and functional impairments (Haskins et al., 2012; Radomski et al., 2016). To do so, OTs can assess individuals with TBI using dynamic assessments of everyday activities (Coelho et al., 2005; Missiuna, 1987), like the Instrumental Activities of Daily Living (IADL) Profile (Bottari et al., 2010a), the Perceive, Recall, Plan and Perform System of Task Analysis (Chapparo & Ranka, 1996) or the Dynamic Lowenstein Occupational Therapy Cognitive Assessment (Katz et al., 2012). Described as an interactive approach between the therapists and clients, this type of assessment allows therapists to explore the effect of various factors (e.g., assistance during the task, task modification, and potential interventions) on the person's performance (Coelho et al., 2005). Through this interaction, therapists may try various types of assistance to identify the ones that should optimally be integrated into the client's routine to improve performance and engagement in meaningful activities (Haywood & Lidz, 2006; Haywood & Miller, 2003) while allowing the client to do as much of the activity on his/her own. This type of assessment contrasts from more diagnostic evaluation tools more specifically focused on documenting the type and frequency of incurred errors. In dynamic assessments, assistance is provided to help the client progress in the task, thus allowing observation of further task components and abilities that could not be observed otherwise (Bottari et al., 2010a). To attain this objective, therapist–client interactions (whether they aim to provide assistance or not) must be in accordance with the person's true needs only if and when necessary, without overestimating or underestimating the person's abilities. This requires the therapist not to think through the activity in place of the person with TBI.

Le Dorze et al. (2014) conducted one of the first detailed studies describing the interactions between an OT and clients with TBI when the aim of the evaluation was to provide the least possible amount of verbal assistance to reflect the person's true needs. The authors described various types of verbal assistance that could be offered during dynamic assessments, including cueing or suggesting a strategy. For this study, verbal assistance was described as interactions (e.g., questions, bits of information provided, and encouragement), “a form of supported thinking”, aiming to help clients think through problems they are confronted with and compensate cognitive limitations (Le Dorze et al., 2014, p. 12). To do so, assistance was offered in a manner that is interactive and personalized to the person's specific cognitive deficits and difficulties in performing daily activities, and not in a standardized fashion. To date, however, verbal assistance in the literature is mostly provided in an intervention context, whether through technology or by a person and using predefined sentences (O’Neill et al., 2018; Thomas & Marsiske, 2014; Wang et al., 2019). Moreover, when focusing on the evaluation context, little is known about how assistance should be enhanced or reduced over time within a session to determine the minimum amount and type of assistance required to meet a client's specific needs. To our knowledge, no study has yet shown on which basis therapists decide, during an evaluation, when it is necessary to provide such assistance, nor when they consider it to be preferable to let the person attempt to formulate goals/plan/detect or correct errors on his/her own to identify how much of the activity he/she can manage on his/her own.

As a result, gathering data that would help us better understand how to provide minimum verbal assistance is needed to improve occupational therapy dynamic assessments with this clientele and further inform personalized treatment planning. One manner in which this problem can be addressed is to explore the clinical reasoning of expert OTs, experienced in offering minimal assistance to clients with TBI during a dynamic assessment. In fact, providing verbal assistance is an interactive process that relies on therapists’ judgment (Le Dorze et al., 2014), as well as on the therapists’ ability to provide assistance online when needed. For this study, clinical reasoning was defined as “reflexive thinking associated with engaging in a client-centred professional practice” (p. 211) including planning before meeting the client, interacting with the client and their caregivers, and professional judgement and knowledge (Unsworth, 2011).

More specifically, the objectives of this study were to determine: (1) How verbal assistance is provided and progressed by OTs with a high level of expertise in evaluating the impact of cognitive deficits on everyday activities in a TBI population using personalized minimal assistance during a dynamic assessment, and (2) why assistance is provided in this manner throughout the evaluation.

## Methodology

### Study Design

An exploratory multiple-case study inspired by the methodology of Yin (2003) was conducted using a post-positivist perspective and an inductive approach. This design was chosen as it allows a detailed understanding of a contemporary phenomenon (i.e., the clinical reasoning of OTs when providing verbal assistance) within its context (i.e., during a dynamic assessment with a specific TBI patient) (Yin, 1994, 2009). As a result, both phenomenon and context were strongly considered throughout the study. Furthermore, contrary to more explanatory research designs, an exploratory design was preferred for the present project as little is yet known in the domain. This study was approved by the ethical review board of the Centre for Interdisciplinary Research in Rehabilitation of Greater Montreal and all participants provided their informed consent.

### Participants

Three OTs (CB, CL, and MT) experienced with administering a dynamic assessment, the IDAL Profile (Bottari et al., 2010a), and working with TBI clients in rehabilitation centers (at least 10 years at the moment of the interview) were selected. The three OTs invited to participate in this study participated in a prior study on the reliability of the IADL Profile (Bottari et al., 2010a). Participants from this previous research were TBI clients who had been referred by one of various specialized TBI programs in the province of Quebec. To aim for replication, OTs were specifically chosen as they provided verbal assistance in a similar context (during the administration of the IADL Profile), received similar training (i.e., three-day workshop and observations of 5–10 evaluations administered by the tool developer) and had the opportunity to master their clinical reasoning skills during training. Moreover, as videos used in the present study were selected from those of previous reliability studies (Bottari et al., 2009a, 2009b), only OTs that had been videotaped when assessing a TBI client using the IADL Profile were considered.

For this study, all participants were presented a video of their own evaluation, as only the OT that administered the evaluation can report his/her own clinical reasoning. Since the administration of the IADL Profile may take up to 4 h, it was decided to use only one video per OT to ensure an in-depth understanding of their clinical reasoning when they provided assistance to their client. Videos were selected from a database of more than 100 videos in collaboration with CB to ensure TBI participants observed during the IADL Profile were all of the same gender and similar age and required substantial verbal assistance during the evaluation for at least four of the eight evaluated tasks. Each OT had previous opportunities to master the IADL Profile through practice before the chosen administration of the evaluation tool (33 times for CB, 5 times for CL, and 6 times for MT).

### Procedures and Data Collection

The IADL Profile evaluation context was used in this study to explore how and why verbal assistance is provided to TBI clients. This tool was specifically developed to determine the level of independence and assistance needs of individuals with TBI in complex everyday activities carried out in the person's home and community environment and is considered as a dynamic assessment (Bottari et al., 2009a, 2009b, 2010a, 2010b). By providing minimal assistance during various complex everyday activities (e.g., receiving guests for a meal, making a budget, and obtaining information), therapists assess a person's level of independence and assistance needs (i.e., physical or cognitive). In the context of this evaluation and in line with its theoretical underpinnings, assistance must be offered only if the client's safety or ability to progress in a task is at stake, as the goal of the evaluation is to identify the extent to which the person is able to think through (formulate goals, plan, problem-solve, detect and correct errors) and carry out a given everyday task on his/her own. It is the premise of the IADL Profile that failure to give the person optimal time and opportunity to think through a given task prior to the therapist providing assistance increases the risk of either underestimating (as assistance may be provided too hastily) or overestimating (as informal interactions may help the person progress without being considered as assistance) both the person's ability and his/her assistance needs. Trained therapists are therefore taught to not talk during the evaluation unless needed.

The study included two steps. An interview was first conducted with each OT by the fourth author (HK) to explore how and why they provided verbal assistance when administering the IADL Profile with a TBI client. Throughout data analysis, preliminary results were also validated and enriched during two focus groups to ensure an accurate understanding of the data. These focus groups provided an opportunity for us to delve more deeply into therapists’ clinical reasoning by allowing them to compare and discuss each other's clinical reasoning in each of their respective contexts. Moreover, the use of both individual interviews and focus groups provided complementary information, as individual interviews provided a more detailed and concrete description of each participant's clinical reasoning, while focus groups likely provided a more contextual and wider understanding of the phenomenon (Lambert & Loiselle, 2008). The focus groups each lasted 1 h and were conducted by the first and fourth authors (MGR and HK).

#### Step 1: Interviews

While watching the videos of their own evaluation, participants were invited to explain (1) how they provided and progressed verbal assistance during the IADL evaluation and (2) why each moment of assistance was provided as such. During the interview, the technique of “think-aloud” was used. This methodology is effective when the goal is to access a person's clinical reasoning (Durning et al., 2013; Ericsson, 2006; Fonteyn et al., 1993). During the interview, comments such as “What were you thinking about?” or “Tell me more about the reason…?” were provided by the interviewer. To familiarize the participants with this technique, a practice was carried out beforehand using a short video. Although participants viewed videos of evaluations they had administered almost 10 years prior, all were able to explain their clinical reasoning during their viewing of the evaluations and critique how verbal assistance was provided according to their actual level of experience. Entire sessions of “think-aloud” interviews were videotaped to facilitate data analysis. Each session lasted a maximum of 4 h.

#### Step 2: Focus Groups

Two focus groups with all three participants (CB, CL, and MT) were conducted to validate and enrich preliminary results obtained during the analysis of data pertaining to each case. A first focus group was conducted to present themes emerging from the two first cases (i.e., CB and CL interviews) using a visual representation of results. Following analysis of the third case (i.e., MT interview) and the first focus group, a second focus group was conducted to review themes emerging from the data analysis process. Through this validation process, emerging themes were validated by all participants and compared with new themes emerging from the focus groups. Both meetings were audiotaped to facilitate data transcription and analysis.

### Data Analysis

All interviews were transcribed, including the clients’ behaviours and difficulties observed in the videos of the IADL Profile evaluations as well as the OTs’ associated think-aloud interviews. A qualitative thematic analysis (Braun & Clarke, 2006) using an inductive approach was then completed. The thematic analysis involves a constant moving backwards and forwards between the data set, the coded extracts and the analysis of the data that are being produced (Braun & Clarke, 2006). This iterative analysis was characterized by six stages: (1) familiarizing with the data, (2) generating initial codes, (3) developing new themes, (4) reviewing themes, (5) defining and naming themes, and (6) producing a report. As proposed by Drapeau (2004) to ensure fidelity and internal validity, an initial coding of all interviews was independently completed by two authors (SBR and HK) to identify preliminary codes. Codes were then compared between both authors to obtain consensus. Emerging codes were also discussed with the first author (MGR). Codes were reviewed and discussed by three authors (SBR, HK, and MGR) to develop new themes and define them. All preliminary results were also presented and discussed with one of the participants and members of the research team (CB) before being validated in the focus groups by the participants using a matrix and visual representation of codes. Finally, each focus group was transcribed and analyzed using the same thematic analysis approach to identify emerging codes and further themes.

## Results

### Participants

All three OTs (MT, CL, and CB) were considered experts at the time of the study. The level of clinical experience with TBI clients was, respectively, 10, 15, and 20 years of experience with the clientele. They all had training on the administration of the IADL Profile, and more specifically on how to provide verbal assistance. Each OT also had the opportunity to administer and master the IADL Profile multiple times before completing the “think-aloud” interviews in this study, as all three participants frequently used the tool in their clinical or research practices.

Although all three OTs were previously involved in the validation of the IADL Profile (Bottari et al., 2010a), CB played a major part in this process as she was the main developer of this observation-based ecological tool. In total, videos of three severe TBI clients were used in this study. TBI clients varied in terms of difficulties observed during the ecological assessment, their level of independence and their living situations. Their characteristics are presented in Table 1.

**Table 1:** Characteristics of the TBI Participants Tested by Each Occupational Therapist

Evaluator of the TBI participant	CB	CL	MT
General characteristics of the TBI participant			
Age, years	19	22	18
Gender	Male	Male	Male
Education level, years	11	14	8
Description of TBI			
TBI severity	Moderate	Severe	Severe
Cause of TBI	Car accident	Car accident	Sport accident
Glasgow Coma Scale at emergency (of 15)	13	7	5
Posttraumatic amnesia, days	N/A	90	N/A
Time postinjury, months	3.5	10	17.75
Functional characteristics			
Living arrangements	House with his parents	Residential resource	Apartment
Rehabilitation status	Outpatient rehabilitation	Outpatient rehabilitation	Outpatient rehabilitation
Tasks requiring verbal assistance during the “evaluation”^1^	Tasks 2–4 and 7, task 8 not completed due to lack of time	Tasks 2, 4, and 6–8	Tasks 2, 4, 7, and 8
Person present at the time of the evaluation	None	Contact person	Father

^1^ Numbers to the following tasks: 1 = putting on outdoor clothes; 2 = going to the grocery store; 3 = shopping for groceries; 4 = preparing a hot meal for guests; 5 = having a meal with guests; 6 = cleaning up after a meal; 7 = obtaining information; 8 = making a budget.

### How Verbal Assistance is Provided and Progressed During an IADL Evaluation

**Figure 1.**
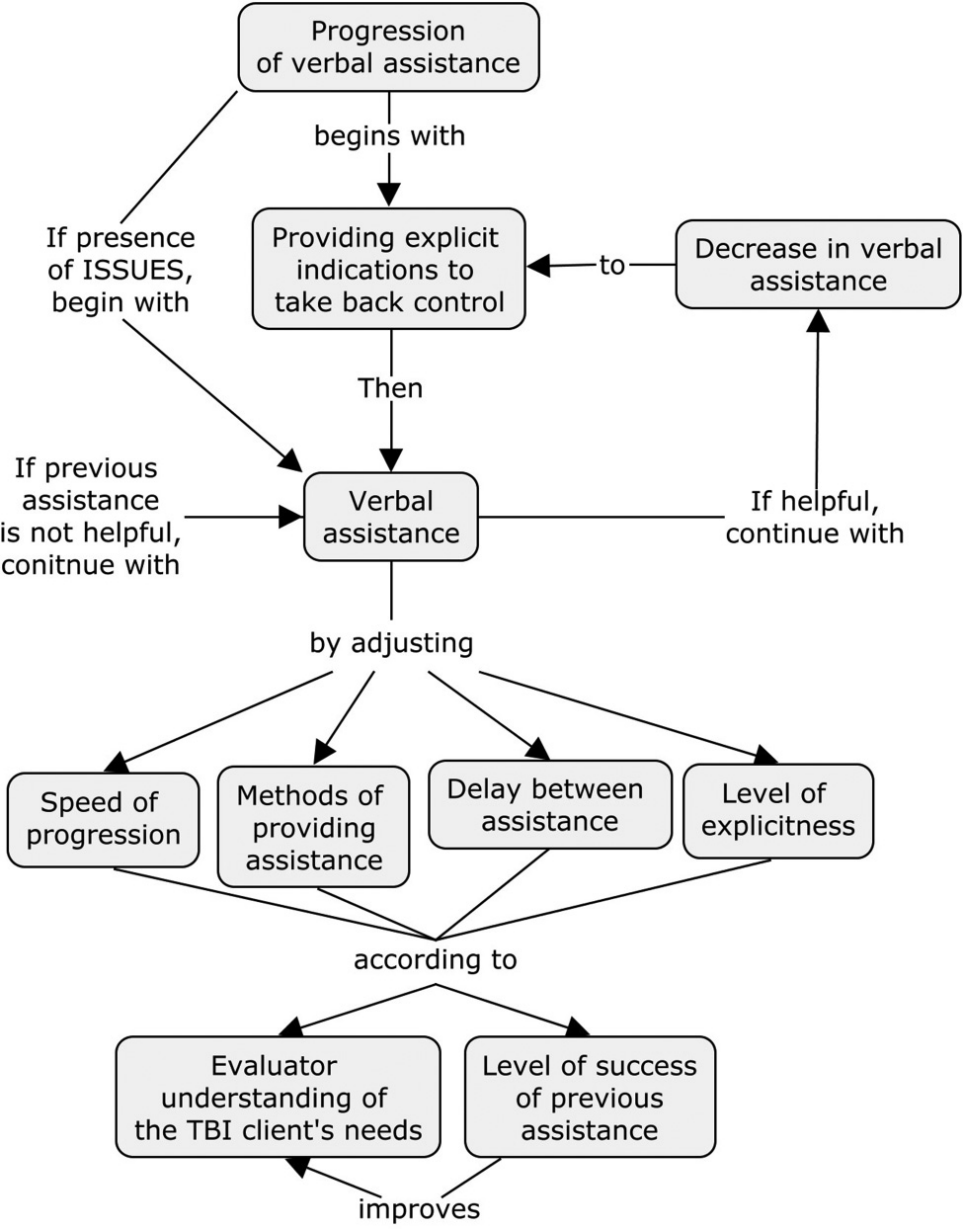
Progression of verbal assistance during the IADL Profile with a TBI client.

Providing and grading verbal assistance throughout the evaluation was described as a flexible process by all three OTs (see Figure 1 for an overview). Before even proceeding to provide assistance, all participants explained that they first began by giving more and more explicit indications to their client to suggest that they take back the control of the task to reduce their reliance on the evaluator. This is illustrated by the following comment by CB: “I am trying to remove myself a little and to remind him that if he forgot something, he has to manage the situation all by himself.” More specifically, OTs mentioned that they used sentences such as “Do as much as you can by yourself” or “Do as if I wasn't here.” However, as was the case for our participants, this type of interaction may not always be helpful. As a result, OTs have to provide graded verbal assistance by varying the methods used to provide the assistance, the speed of progression, delays between each assistance provided, and the level of explicitness of the cues. Finally, OTs highlighted that when previously provided verbal assistance was found to be helpful, assistance that was subsequently used in response to new emerging difficulties tended to retrograde toward earlier used less structured and less explicit assistance to allow their clients to regain some control of the task at hand. The themes are further presented in Table 2.

**Table 2:** Themes Describing how Verbal Assistance was Provided and Progressed by Each Occupational Therapist During the IADL Profile with a TBI client

Themes	Definition and examples	CB	CL	MT	Focus groups
*Exploration of best methods of providing verbal assistance to help their client*	When providing assistance the evaluator will try various methods according to the client's difficulties to help him/her progress in his thinking. Depending on their level of success, the evaluator may choose to continue using such methods or explore other possibilities.	X	X		X
*Adjusting delays between verbal assistance and the speed at which it is progressed over time*	During the evaluation, the evaluator will adjust the delays between each moment of assistance and the speed of progression according to the client's difficulties. In the beginning, the delays will be longer, and they will progressively reduce over time as the evaluator's understanding of the client's needs improves.	X	X	X	X
*Providing more explicit and directive assistance over time*	When having to progress the level and type of assistance, the evaluator will provide more explicit information and/or will be more directive (e.g., use of action verbs) over time to help the client with TBI progress in the task.	X	X	X	
*Reducing assistance over time*	After having provided verbal assistance, the evaluator will reduce the amount and type of assistance over time until he eventually gives the client back the control of the task. This process is intended to provide the least amount of assistance in a personalized manner.	X	X		X

### Why Verbal Assistance is Provided and Progressed in This Manner

Three themes emerged regarding factors influencing the clinical reasoning of OTs when providing verbal assistance. These themes were: (1) factors leading the evaluator to provide assistance, (2) factors leading the evaluator to interact without providing assistance, and (3) factors influencing the progression of the verbal assistance provided by the examiner throughout the evaluation. Definitions of each factor are presented in Table 3.

**Table 3:** Factors Influencing the Clinical Reasoning of Occupational Therapists When Providing Verbal Assistance During the IADL Profile with a TBI Client

Factors	Definition	CB	CL	MT	Focus groups
*Presence of safety and/or emotional issues*	The presence of issues (e.g., fatigue, emotional overload, unsafe behaviors, and frustrations that could lead to stopping the task) leads the evaluator to provide assistance to ensure physical and emotional safety to the TBI client.	X	X	X	X
*Lack of progress in the task*	The TBI client has difficulty to progress and complete the task, which may be observed with great delays, inaction in the task, incomplete thought processes and inappropriate actions according to the evaluation context, all of which may be exacerbated by important cognitive difficulties. As a result, the evaluator must provide assistance to help the person progress and avoid failure.	X	X	X	X
*Requests for help*	The TBI client asks for help (e.g., by asking a question, by looking at the evaluator for help), which leads the evaluator to provide minimal assistance to help the person return to the task.	X	X	X	X
*Off-task discussions*	The TBI client talks about unrelated topics. As a result, the evaluator may decide to him/her pursue these off-task discussions as a way to explore potential ideas, before bringing him/her back to the task.	X	X	X	
*Need to promote the person's engagement in the task*	Throughout the evaluation, the evaluator ensures the therapeutic alliance by encouraging and promoting the good ideas and efforts of the TBI client, and making him/her feel their presence through informal interactions.	X	X	X	
*Need to understand the person's plan*	To better understand the TBI client's difficulty, the evaluator explores his/her plan and comprehension of the evaluation. The evaluator thus asks questions about the client's plan and how he/she will attain the objectives.	X		X	X
*Time available to complete the evaluation*	The amount of time available to complete the evaluation may influence the progression of verbal assistance, as the evaluator may lack the time required to provide only minimal assistance throughout the evaluation.	X	X		
*Level of experience of the evaluator with the clientele and evaluation tool*	The evaluator's level of experience (e.g., number of years of experience with the clientele or with the IADL Profile) can influence how he/she provides assistance during the evaluation.		X		X
*Improved understanding of the person's abilities and difficulties*	Throughout the process, the evaluator develops a better understanding of the person's difficulties and strengths by analyzing their patterns of behaviors and their answer to the methods of assistance previously provided. This understanding thus influences how assistance is provided afterwards.	X	X	X	X
*Familiarity of the person with TBI with the context and the task to be completed*	The TBI client's knowledge (e.g., a person who has never lived alone) and level of familiarity with the task (e.g., meal preparation) and the physical environment (e.g., the cooking area) can influence the amount of assistance they need during the evaluation.	X		X	X

#### Factors Leading the Evaluator to Decide to Provide Verbal Assistance

Four factors were identified by all three OTs as leading them to decide to provide verbal assistance during the evaluation: “presence of safety and/or emotional issues”, “lack of progress in the task”, “requests for help,” and “off-task discussions.”

All three OTs described the presence of safety and/or emotional issues (e.g., fatigue, unsafe behaviors, and increasing frustration), as well as potential unrepairable breakage of equipment, as a reason to provide or upgrade assistance during the evaluation. For example, MT explained that she provided assistance to her client as “he was abandoning the task, he was presenting signs of fatigue, of impatience and irritability.” On the other hand, when such issues were not present, the OTs preferred to let their client continue the task without help, as described by CL: “It was a solution that may not be the first we think of, but for me, it was not unsafe or unusual. […] It's for that reason that he was not given any help.”

The client's ability to progress in the task was also identified by all three OTs as influencing how verbal assistance was provided and progressed during the evaluation. The lack of progress observed during the evaluation lead MT to provide explicit assistance to her client due to his difficulties, as she described here: “It is really explicit. […] I was doing the task for him because he wasn't progressing in his thinking.” OTs also provided assistance to help correct some mistakes interfering with the progression of the task at hand, as described here by CB: “I will try to help him to see if he will realize his miscalculation. He just looked at it, told me what he’d done, but he didn't realise his mistake by himself.” However, when the person was able to progress and stay actively engaged in the task, such as by proposing potential ideas, the OT preferred to let them do as much as possible on their own, as illustrated by CB: “I like to let them try to make sense of their ideas and not intervene. […] He is searching, he is exploring, he is trying to identify ideas. I don't see the need to start helping.”

During the interviews, OTs highlighted the importance they gave to maintaining their therapeutic relationship with their client. As a result, the person's requests for help were identified as a factor leading the OTs to provide assistance during the evaluation. For example, CB explained: “I began to see the pattern. […] He seemed to respect that I didn't help him, but […] when he was no longer able to continue, he looked at me and he knew that I would give him a helping hand.” It should, however, be noted that OTs first reminded the person to do as much as possible on his own.

Finally, OTs (CB, CL and MT) mentioned moments when the client was having off-task discussions, talking about unrelated topics. When faced with these situations, the therapists could decide to immediately bring back her client's attention to the task right away. For example, MT explained that when her client started asking if he/she could play pool, she brought him back to the task by reiterating the evaluation context and the goal of the evaluation. In addition, the OTs explained that they may bring their client's attention back to the task faster if their client had already previously shown off-task behaviors on several occasions. In other cases, the OTs decided to let their client pursue these off-task discussions for some time before bringing their attention back to the task as this was thought to potentially help the client find new solutions, as described by CB: “Sometimes, to let them ramble helps them find answers to their problems.”

#### Factors not Leading the Evaluator to Provide Verbal Assistance

When discussing verbal assistance, all OTs mentioned situations when they found it necessary to talk with their client, but not necessarily to provide assistance. These situations included promoting the client's engagement in the task and trying to understand the client's plan.

To maintain their clients’ compliance and promote their engagement in the task, all three OTs mentioned that they had to encourage and promote the good ideas and efforts of the client as the latter may be experiencing a difficult situation. For example, CB explained: “I tried to maintain a therapeutic alliance. This is someone with difficulties […] so I think it is important to enhance what [her client] does well.” The need to make the client feel the therapist's presence during the evaluation by having informal interactions was also mentioned by the OTs. For example, CL explained that her “interventions [were] more informal to reassure [her client]” during the evaluation.

Finally, OTs (CL, MT, and focus groups) identified the need to better understand the client's plan of action and comprehension of the evaluation context, as it helped them form a better understanding of the person's difficulties. The OT thus, in keeping with IADL Profile administration guidelines, initially questioned their client's plan to better grasp his/her understanding of the task to complete. Moreover, when the client's expressed plan deviated from the initial instructions provided (e.g., the client's plan did not include going to the grocery store to buy necessary ingredients to prepare a meal as requested in the instructions provided by the examiner), the OT again questioned their client to either challenge his/her plan or check if assistance was required to clarify the context. By providing this assistance, the occupation therapists were able to observe their client in a situation that could not be observed otherwise (e.g., moving around in the community to go to the grocery store). Such exploration was also helpful as OTs could compare the execution to the previous plan. For example, CL tried to make her client rethink his plan so that he could see that his idea was not congruent with the evaluation context. OTs, however, reiterated during the second focus group that verifying the client's plan and comprehension of the evaluation context was included as a first step in the administration of the IADL Profile and should consequently not be considered as verbal assistance. Nonetheless, when further investigation of the client's comprehension and plan was required (including the need to challenge them), it could be considered as verbal assistance and this assistance should be provided in a manner that offers the least amount of added information.

#### Factors Influencing how Verbal Assistance is Provided and Graded Throughout the evaluation

Finally, four factors emerged as influencing how verbal assistance is provided and graded throughout the evaluation (as presented in Figure 1): “Time available to complete the evaluation,” “level of experience of the OT with the clientele and evaluation tool,” “improved understanding of the person's abilities and difficulties,” and “level of familiarity of the person with TBI with the context and the tasks to be completed”).

Time available to complete the evaluation was a contextual limit identified by two of the OTs as influencing how they provided and graded assistance during the evaluation. Despite the flexibility, the IADL Profile allows, OTs mentioned having to increase the assistance they provided to ensure that the evaluation was completed within an acceptable timeframe for both the client and the therapist. For example, CB explained, after the client having been given 3 h to complete the evaluation: “I had no time left, I had a time constraint. So, I was giving explicit assistance right away.” This limit was, however, only observed during the last task of the eight included in the IADL Profile (i.e., making a budget), as the client had been provided with a large amount of graded assistance throughout the evaluation prior to this. On the other hand, when beginning the evaluation that limit was not generally experienced, as illustrated by CL: “I don't help because I want to validate my hypotheses. […] There is nothing that compromises his safety, there is time available.”

Their own level of experience with the clientele and the evaluation tool was also identified as the main factor by the OTs (CB and focus groups). During the first focus group, the OTs explained how administering the tool efficiently and providing personalized assistance at the same time may be challenging for new graduates as “They will try to write things down […] to be able to think after [the evaluation]. We can expect that it would be very different [for] someone with a lot of experience.” Moreover, experience with the clientele may affect the OT's clinical reasoning, as explained by CB: “It seems that when we are [practising therapists], we lose our points of comparison regarding what is pathological because all we see are people who have had a head trauma, who are slow [and have] cognitive difficulties […]. We adapt ourselves to their difficulties and somehow, we become a less severe judge.” As a result, she mentioned she could have provided assistance more rapidly with her client as he demonstrated significant difficulty progressing in the task.

How verbal assistance is provided and graded during the evaluation was also influenced by the OT's improved understanding of the client's abilities throughout the evaluation process, as mentioned by all three OTs. In fact, by offering verbal assistance over a certain period of time with any one client, OTs were able to identify “patterns of difficulties.” For example, as the client of CL was being impulsive, she explained: “I find that this fits with the [pattern of difficulties] observed from the beginning of the evaluation with this client.” Using this understanding, the OTs may be able to better identify the client's assistance needs: “There are some difficulties that will be repeated, so […] we seek to understand if they are explained by the same underlying problem. Are they helped when the therapist provides the same types of assistance or not?” (FG1).

Finally, the level of familiarity of the client with the context and the tasks being completed as part of the evaluation was identified as influencing the clinical reasoning of OTs (CL, MT, and focus groups) when providing verbal assistance during the IADL evaluation. For example, MT was less severe in her evaluation even though she provided explicit cues as her client was not familiar with cooking: “It probably denotes that he is not used to cooking because he does not know how many millilitres are in a cup […]. I gave him pretty explicit assistance because he does not seem to be familiar with [cooking].”

## Discussion

This multiple-case study aimed to explore the clinical reasoning of three OTs when providing verbal assistance to a TBI client during a dynamic IADL evaluation. More precisely, this study aimed to explore (1) how verbal assistance was provided and progressed by these three experts as well as (2) why assistance was progressed in this manner. According to our results, progressing verbal assistance during a dynamic IADL evaluation is a flexible and nonlinear process, involving various adjustments (e.g., types of assistance, level of explicitness, and speed of progression) according to the client's observed behaviors and difficulties. Although verbal assistance was most often graded from less to more explicit, all three OTs highlighted the need, when possible, to limit their offer of verbal assistance and their involvement in the task to let the clients do most of the thinking on their own. Moreover, various elements observed during the evaluation (e.g., lack of progress in the activity and off-task discussions), as well as factors related to the context, the evaluator and the client, influenced how OTs provided and progressed verbal assistance during the dynamic IADL evaluation.

As mentioned previously, providing and grading verbal assistance was described as a flexible and nonlinear process. This approach is consistent with dynamic assessments, as strategies (i.e., verbal assistance) were provided in a nonstructured manner to help the client progress in the task and therefore attain a better performance (Coelho et al., 2005; Haywood & Tzuriel, 2002). In fact, in line with the IADL Profile's ultimate goal of assessing the true level of independence of the client, the flexible progression of verbal assistance allowed the OTs to not only explore the clients’ abilities by observing their thinking and behaviors, but also to characterize their assistance needs (e.g., types of assistance to prioritize) (Bottari et al., 2010b). As a result, the OTs were able to identify potential personalized strategies that could be put into place in an intervention plan to support activities that are otherwise not adapted to the client's residual abilities. This is not surprising, as dynamic assessment was previously described as bonded to intervention (Katz et al., 2012; Lidz, 1992) and was found to guide more efficient intervention plans than conventional assessments in clients with unilateral neglect (Toglia & Cermak, 2009). The ability of therapists to explore and identify the best types of verbal assistance for their clients is even more beneficial as cognitive-communication disorders, which are difficulties in communication due to underlying cognitive deficits (e.g., attention and executive functioning), are common following a TBI (Togher et al., 2014) and could potentially impact the person's ability to interpret and understand provided assistance. Finally, the three OTs emphasized the need to limit the level of assistance they provided to their client when possible, which is congruent with IADL Profile administration guidelines (Bottari et al., 2010b) and previous work from Le Dorze et al. (2014).

Various factors influencing how verbal assistance was provided and progressed throughout the evaluation were also identified in this study, going beyond results obtained in the study by Le Dorze et al. (2014). Consistent with the person–environment–occupation model in occupational therapy (Law et al., 1996), the process of providing verbal assistance was influenced by the client's abilities (as observed during the evaluation and the presence of issues such as a lack of progression and asking for help), the context (including the characteristics of the evaluation and the evaluator) and the demands of the tasks to be completed. These holistic factors also influenced the clinical reasoning of the OTs. As previously mentioned, providing verbal assistance was a complex and iterative process involving various adjustments and explanatory factors over time. Due to its complexity, such processes seemed to involve multiple levels of clinical reasoning, which is coherent with previous studies (Fleming, 1991; Unsworth, 2004, 2011). In fact, Unsworth (2004) conceptualized clinical reasoning in a client-centered practice by including two levels of reasoning: pragmatic reasoning, which encompasses the impact of the environment on the reflexive process (i.e., contextual factors identified in this study such as the time available for the evaluation); and narrative/scientific reasoning, which is composed of procedural reasoning (i.e., what are the hypotheses in terms of difficulties and assistance needs, and how to test them), interactive reasoning (i.e., how to communicate and understand the client, including when to promote the client's engagement through encouragement) and conditional reasoning (i.e., how better understanding the actual and potential abilities of the client influences the next interventions and assistance to offer). Furthermore, both are influenced by the therapist's worldview, which involves the therapist's abilities and attitudes (i.e., factors related to the evaluator such as the level of experience with the clientele and the administration tool). Interestingly, while most themes emerging from this study were mentioned by both CB and CL, four of the themes were not mentioned by MT. This could be explained by a variability in the ability to explicit tacit knowledge (i.e., know how), even among experts. Nonetheless, using this understanding of how clinical reasoning plays a major part in the process of providing personalized verbal assistance and how such a process could potentially be used in the intervention, better training could be developed to guide therapists throughout the complex process that is involved in progressing verbal assistance with individuals with cognitive deficits so that this assistance allows them to use their residual cognitive abilities to the utmost degree. Ultimately, we hypothesize that with a better understanding of their clients potential and difficulties, therapists could develop intervention plans which could support their clients by meeting their assistance needs in everyday activities.

This descriptive multiple-case study provided a first understanding of the clinical reasoning of OTs when providing verbal assistance during an IADL evaluation, and how verbal assistance may be progressed. Despite the complexity of this process, this study offered a first picture of the role of verbal assistance during a dynamic assessment. Using a rigorous method including independent coding and thorough validation, this detailed case study included three experts who iteratively validated our results. The use of the technique of “think-aloud” was also a strength of this study, as it is known to provide access to therapists’ clinical reasoning (Durning et al., 2013; Ericsson, 2006; Fonteyn et al., 1993). Moreover, as TBI clients were evaluated within their real-world environment, no specific differences can a priori be thought of to exist between the context of the present project and the clinical context, thus facilitating the replicability of these results in clinical practice. However, the generalizability of findings is limited as only three OTs were involved, and only three videos of clients were used to discuss the clinical reasoning of the OTs during the interviews, though each was described in detail by the experts and analyzed in-depth. It should also be noted that the initial study during which reviewed assessments were videotaped was completed 10 years prior to the present one. Nonetheless, the use of video allowed detailed recollection of the assessments while allowing further analysis of the way they provided verbal assistance using their expertise at the time of the interview. Further research with larger samples is thus required to deepen our understanding of how to provide personalized verbal assistance. Research focusing on verbal assistance provided in other contexts (e.g., during the intervention and during other dynamic assessments) are also necessary.

## Conclusion

Although challenging, determining and providing verbal assistance in a minimal and personalized manner allow OTs to obtain a clearer picture of the abilities of individuals with TBI during a dynamic IADL evaluation while promoting the engagement of their clients in the assessment. According to this study, providing verbal assistance when administering a dynamic IADL evaluation with a client is an iterative process requiring strong clinical reasoning on the part of the OT. By exploring the thought processes of three experts, we were able to better understand how verbal assistance was provided and progressed during the evaluation as well as why assistance was provided in this manner. Using these results, verbal assistance could be better characterized according to the person's needs and translated into personalized interventions provided within the home environment. Future research including larger samples is needed to further improve our understanding of verbal assistance, how to better offer it according to the person's needs and abilities, and how to link this process to personalized intervention plans.

## Key messages

- Providing and progressing verbal assistance during an IADL evaluation is an iterative process that requires the OT's clinical reasoning.
- When providing verbal assistance, OTs should begin by encouraging and giving back the control of the task and of the decisions to be made to the person, before progressing toward implicit and then more explicit and directive assistance.
- By providing verbal assistance that is adapted to observed difficulties during an observation-based assessment, OTs can better understand their clients’ abilities and simultaneously, the best methods to assist them.
